# The effect of hydroperiod and predation on the diversity of temporary pond zooplankton communities

**DOI:** 10.1002/ece3.1593

**Published:** 2015-07-07

**Authors:** Marcus Zokan, John M Drake

**Affiliations:** Odum School of Ecology, University of GeorgiaAthens, Georgia, 30602

**Keywords:** Diversity, mesocosm, temporary ponds, zooplankton

## Abstract

In temporary pond ecosystems, it is hypothesized that the two dominant structuring forces on zooplankton communities are predation and demographic constraints due to wetland drying. Both of these forces are deterministic processes that act most strongly at opposing ends of a hydroperiod gradient. Our objective was to test how these two processes affect *α*- and *β*-diversity of zooplankton communities derived from a diverse temporary pond system. We hypothesized that decreased hydroperiod length and the presence of salamander larvae as predators would decrease *β*-diversity and that intermediate hydroperiod communities would have the greatest species richness. Our 1-year mesocosm experiment (*n* = 36) consisted of two predation treatments (present/absent) and three hydroperiod treatments (short/medium/long) fully crossed, seeded from the resting egg bank of multiple temporary ponds. In total, we collected 37 species of microcrustacean zooplankton from our mesocosms. A reduction in hydroperiod length resulted in lower *α*-diversity, with short-hydroperiod treatments affected most strongly. Endpoint community dissimilarity (*β*-diversity) was greatest in the medium-hydroperiod treatment with regard to species presence/absence, but was greatest in the long-hydroperiod treatment when abundances were included. Predation by salamander larvae led to reduced *β*-diversity with respect to species presence/absence, but not among abundant species, and had no effect on *α*-diversity. Our results suggest that environmental changes that reduce hydroperiod length would result in reduced *α*-diversity; however, intermediate hydroperiod length appear to enhance *β*-diversity within a group of wetlands.

## Introduction

**Figure 1:**
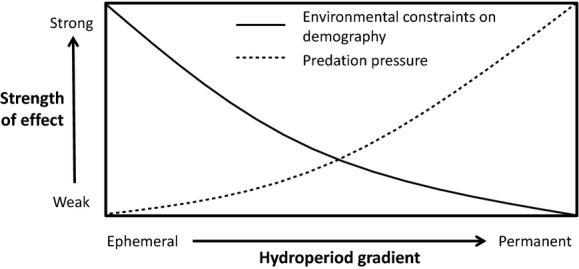
A conceptual diagram of the predation-permanence gradient in temporary wetlands, developed from Wellborn et al. (1996). The strength of the environmental constraint on life history due to wetland drying is greatest when hydroperiod is short and alleviates as hydroperiod increases. On the other hand, the strength of predation is greatest in long-hydroperiod ponds and diminishes in shorter hydroperiod wetlands due to fewer predators capable of sustaining populations in these habitats. The intersection of these two relationships suggests that the pressure exerted by these two processes may be lessened in moderate hydroperiods, although the true shape of these relationships is unknown.

Temporary wetlands are aquatic habitats that dry on some periodicity ranging from ephemeral pools inundated only a few weeks per year to semipermanent ponds that dry completely only during drought. They are notable for their species-rich communities of aquatic invertebrates (Williams et al. 2004), many of which do not occur in permanent waters. The constraint of hydroperiod length (the length of time a wetland holds water) has been established as an important factor limiting the occurrence of species in temporary wetland habitats (Mahoney et al. 1990; Wellborn et al. 1996). If the population of a species cannot produce enough drought-resistant life-stages within a hydroperiod, or be capable of dispersing away, then the species will not persist. Another important factor impacting wetland communities is predation by salamander larvae, which has been noted to have top-down effects on wetland invertebrate communities (Holomuzki et al. 1994; Blaustein et al. 1996). These two factors are central to a conceptual model by Wellborn et al. (1996) termed the “predation-permanence gradient” (Fig.1). This model states that the constraints of hydroperiod length are strongest in wetlands with short hydroperiods (wetlands that frequently dry down), whereas the effects of predation are most important in long-hydroperiod wetlands (wetlands that typically contain standing water and where dry down is infrequent).

According to the model, these two factors determine the presence or absence of species, due to whether species are sensitive to predation or adapted to frequent drying. By definition, predation-sensitive species will not survive in permanent wetlands where predators are abundant, but will survive in the latter, where predators are reduced. Species that inhabit temporary ponds must be adapted to wetland drying. For those that produce resting stages, they must produce them prior to a dry down and in sufficient quantities for their population to survive the dry phase and truncated wet phases. Because wetland drying and predation are considered deterministic processes and act in opposing directions, community similarity should be greatest at both ends of the predation-permanence gradient.

Empirical studies by Chase (2003, 2007) found that community dissimilarity, or *β*-diversity, is greater in permanent ponds than temporary wetlands, suggesting that *β*-diversity increases with hydroperiod length, but that predation increases similarity (decreases *β*-diversity) in permanent wetlands (Chase et al. 2009). Thus, it remains unclear what the net effect of these processes will be when both are acting together. Because predation and wetland drying act most strongly at opposing ends of the hydroperiod gradient and are reduced in intermediate hydroperiods, dissimilarity could be increased at intermediate levels of both. This greater dissimilarity could result from greater species richness or *α*-diversity at some level of intermediate hydroperiod. Because these predictions have not been tested experimentally, we conducted a mesocosm experiment in which predation and hydroperiod were manipulated to test the following hypotheses within temporary wetland zooplankton communities:

- H1 – zooplankton community dissimilarity (*β*-diversity) increases with hydroperiod length. More specifically, we predict that endpoint communities in long-hydroperiod treatments will have greater *β*-diversity among replicates than those in short-hydroperiod treatments. This is because the constraint on life history due to wetland drying is lessened as hydroperiod length increases;
- H2 – zooplankton community similarity increases in the presence of predation. We predict endpoint communities in the treatment exposed to predation by salamander larvae will have lower *β*-diversity among replicates than those in which salamander predation is absent; and
- H3 – intermediate hydroperiods will have the greatest zooplankton species richness (*α*-diversity). We predict endpoint communities in medium-hydroperiod treatments will have the greatest species richness. The pressures of predation and wetland drying are reduced in intermediate hydroperiods, leading to the possibility of greater species richness when both of these pressures are lessened.

The goal of this experiment was to address whether and how the impacts of salamander predation and the demographic constraints imposed by wetland drying reduce a large species pool to the smaller communities observed in natural wetlands. Our results indicate that hydroperiod length and the frequency of drying have important effects on both *α*- and *β*-diversity; however, salamander predation at the densities tested had only minor impacts on the zooplankton community.

## Methods

**Figure 2:**
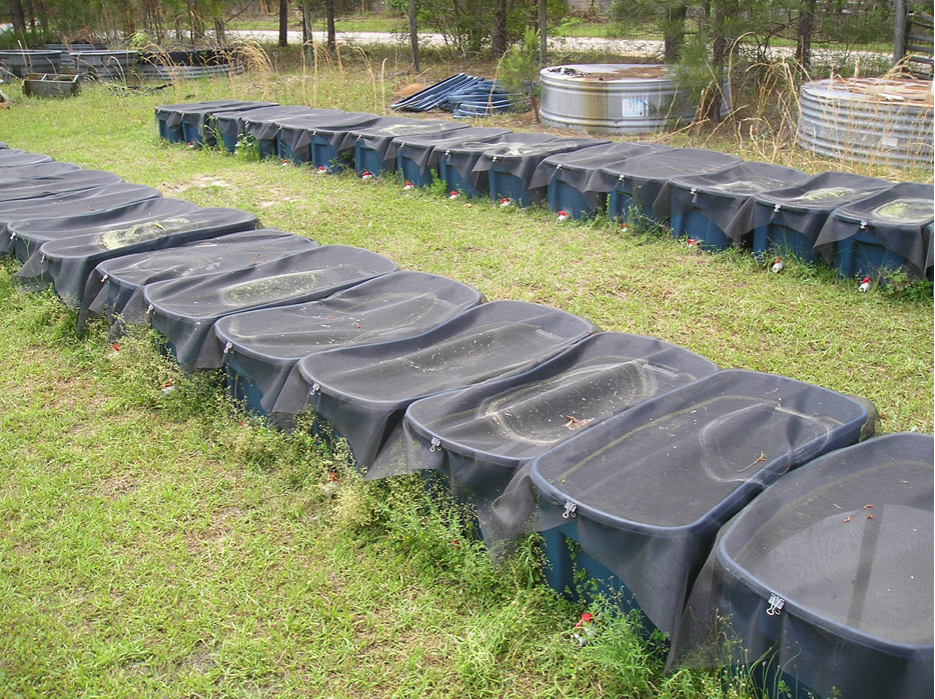
Photograph of the experimental setup. Mesocosms consisted of thirty-six 189-L plastic containers with overall dimensions of 108 cm × 55 cm × 45 cm arranged in two rows of eighteen. Mesocosms were covered with screen and had drainage valves installed near the bottom to manipulate water levels.

The experiment was conducted at the Savannah River Site Ecology Laboratory (SREL), South Carolina, between 19 September 2012 and 4 September 2013. Experimental mesocosms consisted of 189-L plastic containers with overall dimensions of 108 cm × 55 cm × 45 cm (Fig.2). Treatments consisted of three hydroperiod manipulations of different duration (short, medium, and long) and two predation treatments (salamander larvae present vs. absent) in a fully crossed, balanced design with six replicates per treatment. Each mesocosm was seeded with ∼200 g of sediment from each of five nearby wetlands that span a hydrologic gradient from ephemeral pools to semipermanent ponds. The sediments contained the resting stages of zooplankton and other organisms that exist at each of these sites and were mixed and spread among all 36 mesocosms. Using this range of sediment samples, the intention was that each mesocosm would be inoculated with a good representation of the regional species pool. In addition, an 18 L water sample was taken from one wetland, mixed well, and 0.5 L of it was added to each mesocosm to provide a base level of primary and secondary production in anticipation of the addition of salamander larvae. The added water samples were poured through screen to prevent the addition of large macroinvertebrates although smaller instars may have passed through. The impact of predation by macroinvertebrates is not insignificant (Castilho-Noll and Arcifa 2007; Horppila et al. 2009) and although they were not common in this experiment, could be a potential confounding factor. The tops of all mesocosms were screened to prevent colonization by animal-transported plankton species. Treatments implemented on each mesocosm were assigned randomly.

### Hydrology treatments

To simulate environmental dry down, water was released from a drainage valve on each mesocosm and run through a 183-*μ*m mesh plankton net to collect any zooplankton resting stages that were washed out, which were then returned to the mesocosm. Mesocosms were drained at once rather than gradually, as mesocosms could only be visited monthly. While this does not mimic dry down in natural wetlands, data from the source wetlands did not indicate that production of resting stages was closely tied to impending dry down. The short-hydroperiod treatment was inundated for spans of 132, 96, and 64 days (the experimental endpoint). The medium-hydroperiod treatment was initially inundated for 218 days and was refilled for the remaining 92 days till the end of the experiment. The permanent treatment remained wet the entire 350-day duration of the experiment. In simulated dry downs, mesocosms were left dry for at least 28 days before refilling. During the first dry down of the short-hydroperiod treatment, repeated rainfall kept shallow puddles (2–3 cm) within those mesocosms; however, in subsequent dry downs, mesocosms were tipped on their sides to prevent water from entering.

### Predation treatments

Predator treatments involved the addition of (*n* = 2) marbled salamander (*Ambystoma opacum*) or mole salamander (*Ambystoma talpoideum*) larvae. First, larvae of newly hatched *A. opacum* were added at day 132. These were replaced by 3-month-old *A. talpoideum* beginning on day 258 to reflect characteristic seasonal periodicities in their life histories and to maintain the presence of salamander larvae throughout the duration of the experiment. Specifically, *Ambystoma opacum* in SRS migrate to breeding ponds in the fall and larvae metamorphose between April and June (Pechmann 1995). In contrast, *Ambystoma talpoideum* larvae hatch in winter and metamorphose over the summer (Scott 1993). However, many become paedomorphic in wetlands that maintain constant water levels and are present throughout the year (Semlitsch 1987; Pechmann 1995). Thus, the change in species at day 258 (June 4th) mimics the seasonal replacement of species seen in these types of wetlands. The 18 mesocosms in the predation treatment were stocked with two salamander larvae per mesocosm (3.37/m^2^), which is at the low end of natural hatching densities, but is within range of densities present as larvae approach metamorphosis (Scott 1990). When mesocosms were dry, salamanders were removed to a holding tank and fed a diet of zooplankton and insect larvae; they were returned to the mesocosms once the containers were refilled. In natural wetlands, salamander larvae would not be present after a dry down; however, we returned larvae to the mesocosms post dry down to maintain predation pressure throughout the experiment. Salamander density was monitored periodically by sweeping a dip net through each mesocosm until all were accounted for or three consecutive sweeps failed to produce another individual. Additional larvae were added as needed to maintain the treatment density. Our experimental protocol was in accordance with the procedures of and approved by The University of Georgia Institutional Animal Care and Use Committee.

### Sample collection

One zooplankton sample per mesocosm was taken monthly using a tube trap sampler (Paggi et al. 2001). One mesocosm in the short-hydroperiod/no-predator treatment group was damaged and drained out between the penultimate and final sampling day and was removed from all analyses for that date. Cladocerans, cyclopoid copepods and calanoid copepods were identified to the species level where possible. Other individuals of Calanoida, Harpacticoida, Ostracoda, and Anostraca were identified to class level and were also counted and designated as pseudospecies. Water conditions (pH, conductivity, temperature) were monitored in conjunction with each sampling using a YSI Professional Plus.

### Statistical analysis

To examine the effect of experimental treatments on community similarity, the abundances of each species present on the final sampling date were converted to a community matrix. Following Chao et al. (2012), two pairwise measures of *β*-diversity (Sørensen–Dice index and Morisita’s overlap index) were calculated from each matrix, reflecting dissimilarity in presence/absence and relative abundances, respectively. These measures can be derived from the classical definition of *β*-diversity and are themselves transformations of a single diversity metric, but with different weights given to species frequencies (Jost et al. 2011; Chao et al. 2012). The Sørensen–Dice index represents differences in species presence (*q* = 0) and the Morisita’s overlap index represents differences in dominant species (*q* = 2) in the Hill number diversity framework (Jost et al. 2011). The Sørensen–Dice index and Morisita’s overlap index were calculated using the “vegan” package in R. To compare differences between treatment groups, we used Welch’s ANOVA due to unequal group sizes and heteroscedasticity; ANOVA comparisons used within-treatment *β*-diversity. Pairwise differences between hydroperiod treatment groups were tested using a post hoc Games–Howell test. Statistical tests were performed in R 3.2.0, R Foundation for Statistical Computing, Vienna, Austria.

Sample species richness was calculated for each sample as total number of species and pseudospecies, omitting immature and male cyclopoids. The lower bound of true species richness was estimated using the bias-corrected Chao1 estimator (Chao et al. 2005). Two additional measures of *α*-diversity, estimators of Shannon’s index (Chao and Shen 2003) and the Simpson index (minimum variance unbiased estimator) (Magurran 1988) that account for unseen species, were calculated for each sample using abundances of the same taxonomic units used for species richness calculation. Species richness, Shannon’s index, and the Simpson index represent the three levels of *α*-diversity (*q*) recommended by Chao et al. (2012) to characterize a community of species. Species richness reflects the number of species present, but gives no information on species abundances. A higher Shannon’s index indicates a higher number of species in more equal abundances, whereas a high Simpson index value represents a community low in species in which only a few species dominate the total community abundance. These metrics can be converted to Hill numbers, with the interpretation of species richness (*q* = 0) as the number of species, exponential of Shannon’s index (*q* = 1) as the number of average species, and inverse Simpson index (*q* = 2) as the number of dominant species (Chao et al. 2012). The levels of diversity (*q*) in measures of both *α*- and *β*-diversity are equivalent regarding the weights given to species frequencies. Richness and diversity estimates were calculated using the program SPADE (Chao and Shen 2010). Differences in *α*-diversity between treatments for each sampling date were analyzed using Welch’s ANOVA due to unequal group sizes and heteroscedasticity in some comparisons; pairwise differences between hydroperiod treatment groups were tested using a post hoc Games–Howell test.

## Results

Over 40,000 individuals of at least 37 species were collected during this study (Table1), representing 46% of taxa known from the wetlands from which the mesocosm communities were derived. Total mesocosm sample species richness ranged from 12 to 23 species (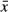  = 16.19, SD = 2.86). The endpoint communities held 19 total species and ranged from 1 to 8 species per mesocosm (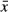  = 3.37, SD = 1.97). There were no species present in the short-hydroperiod treatment that were not present in the other two hydroperiod treatments.

**Table 1:** Table of all zooplankton taxa collected from wetlands from which the communities in the mesocosms were derived. Taxa that have a frequency listed were also collected within mesocosms. Frequency refers to the number of mesocosms a particular species was collected in throughout the duration of the experiment

Species/pseudospecies	Freq.	Species/pseudospecies	Freq.
Anostraca	2	* Bosmina tubicen*	34
* Streptocephalus seali*		* Camptocercus cf. rectirostris*	31
* Eubranchipus stegosus*		* Ceriodaphnia laticaudata*	1
Laevicaudata		* Ceriodaphnia megops*	
* Lynceus gracilicornis*		* Ceriodaphnia cf. dubia*	
Calanoida	1	* Chydorus eurynotus*	
* Agalaodiaptomus atomicus*		* Chydorus linguilabrus*	
* Agalaodiaptomus clavipoides*		* Chydorus* sp. *A*	22
* Agalaodiaptomus stagnalis*	1	* Chydorus* sp. *B*	36
* Hesperodiaptomus augustaensis*		* Daphnia laevis*	6
* Leptodiaptomus moorei*		* Diaphanosoma cf. brachyurum*	36
* Onychodiaptomus sanguineus*		* Disparalona acutirostris*	
Cyclopoida		* Dunhevedia cf. crassa*	
* Acanthocyclops robustus*	15	* Ephemeroporus hybridus*	3
* Acanthocyclops venustoides*		* Eurycercus longirostris*	
* Diacyclops crassicaudis*		* Eurycercus microdontus*	
* Diacyclops navus*		* Grimaldina brazzai*	1
* Diacyclops nearcticus*		* Ilyocryptus bernerae*	1
* Diacyclops thomasi*		* Ilyocryptus gouldeni*	6
* Ectocyclops phaleratus*		* Ilyocryptus silvaeducensis*	24
* Eucyclops elegans*		* Ilyocryptus spinifer*	7
* Eucyclops pectinifer*	25	* Kurzia cf. media*	2
* Macrocyclops albidus*		* Lathonura cf. rectirostris*	
* Macrocyclops fuscus*		* Macrothrix elegans*	31
* Megacyclops cf. viridis*		* Macrothrix cf. spinosa*	11
* Microcyclops* sp.		* Macrothrix sp. B*	
* Orthocyclops modestus*		* Moina micrura*	15
* Paracyclops chiltoni*		* Moinodaphnia macleayii*	
* Thermocyclops parvus*		* Oxyurella brevicaudis*	
* Tropocyclops* sp.	25	* Paralona cf. pigra*	4
Harpacticoida	2	* Picripleuroxus denticulatus*	
Cladocera		* Picripleuroxus stramineus*	
* Acantholebris curvirostris*		* Polyphemus cf. pediculus*	
* Acroperus* sp.		* Pseudochydorus cf. globosus*	
* Alona costata*	31	* Pseudosida bidentata*	27
* Alona guttata*	4	* Scapholebris armata*	12
* Alona manueli*	1	* Scapholebris freyi*	33
* Alona ossiani*	6	* Simocephalus cf. exspinosus*	
* Alona quadrangularis*		* Simocephalus serrulatus*	2
* Alona rustica americana*	1	* Streblocercus pygmaeus*	1
* Alonella excisa*	33	* Streblocercus serrulatus*	
* Alonella exigua*		Ostracoda	22

**Figure 3:**
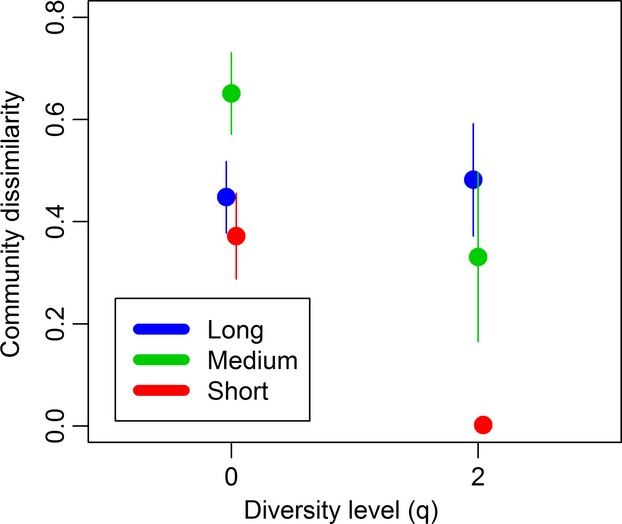
Mean community dissimilarity calculated within each hydroperiod treatment for endpoint communities at two diversity levels, Sørensen–Dice index (*q* = 0) and Morisita’s overlap index (*q* = 2). The colored lines indicate ±2 SE.

*β*-diversity within hydroperiod treatments measured using the Sørensen–Dice index differed between treatments (*F* = 22.10, df = 119.8, *P* < 0.001) and was greatest within the medium-hydroperiod treatment (Fig.3), which differed from both the short (Cohen’s *d* = 1.22, *P* < 0.001)- and long-hydroperiod treatments (Cohen’s *d* = 0.81, *P* < 0.001). The long-hydroperiod treatment was also more dissimilar than the short-hydroperiod treatment (Cohen’s *d* = 0.51, *P* = 0.02). The same analysis performed on Morisita’s overlap index also found differences between treatments (*F* = 86.54, df = 86.68, *P* < 0.001). In this analysis, the short-hydroperiod treatment had greater similarity within treatment than both the medium (Cohen’s *d* = 1.05, *P* < 0.001)- and long-hydroperiod treatments (Cohen’s *d* = 2.26, *P* < 0.001); there was not a significant difference between the medium- and long-hydroperiod treatments (Cohen’s *d* = 0.34, *P* = 0.14).

**Figure 4:**
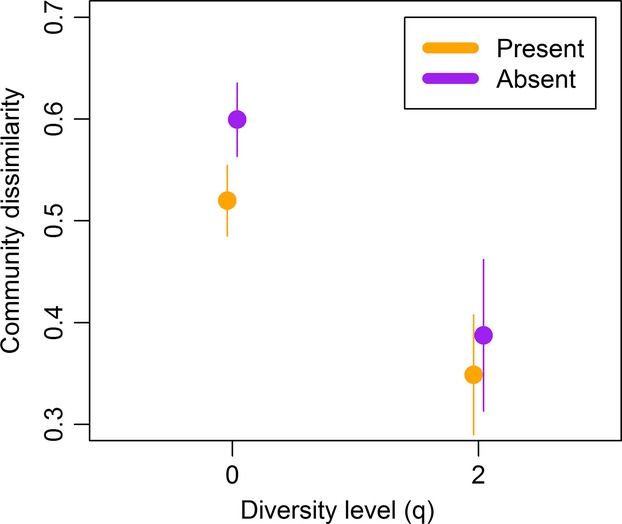
Mean community dissimilarity calculated within each predation treatment for endpoint communities at two diversity levels, Sørensen–Dice index (*q* = 0) and Morisita’s overlap index (*q* = 2). The colored lines indicate ±2 SE.

*β*-diversity within predation treatments differed in Sørensen–Dice index calculations (*F* = 10.09, df = 284.38, *P* = 0.002), with dissimilarity greater within the no-predator treatment than the predator treatment (Cohen’s *d* = 0.38, *P* = 0.002; Fig.4). The Morisita overlap index did not differ between predation treatments (*F* = 0.68, df = 264.64, *P* = 0.41).

**Figure 5:**
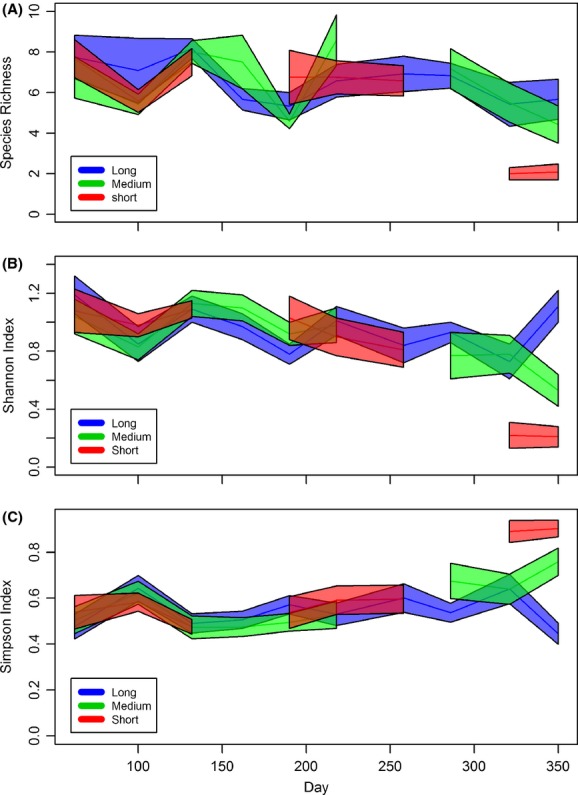
(A) Mean species richness estimated using the Chao1 estimator by hydroperiod treatment, (B) mean estimated Shannon’s index by hydroperiod treatment, and (C) mean estimated Shannon’s index by hydroperiod treatment over the 350-day experiment. The colored polygons enclose regions bounded by ±1 SE. Breaks in the polygons represent periods when the mesocosms within that treatment were dry.

**Figure 6:**
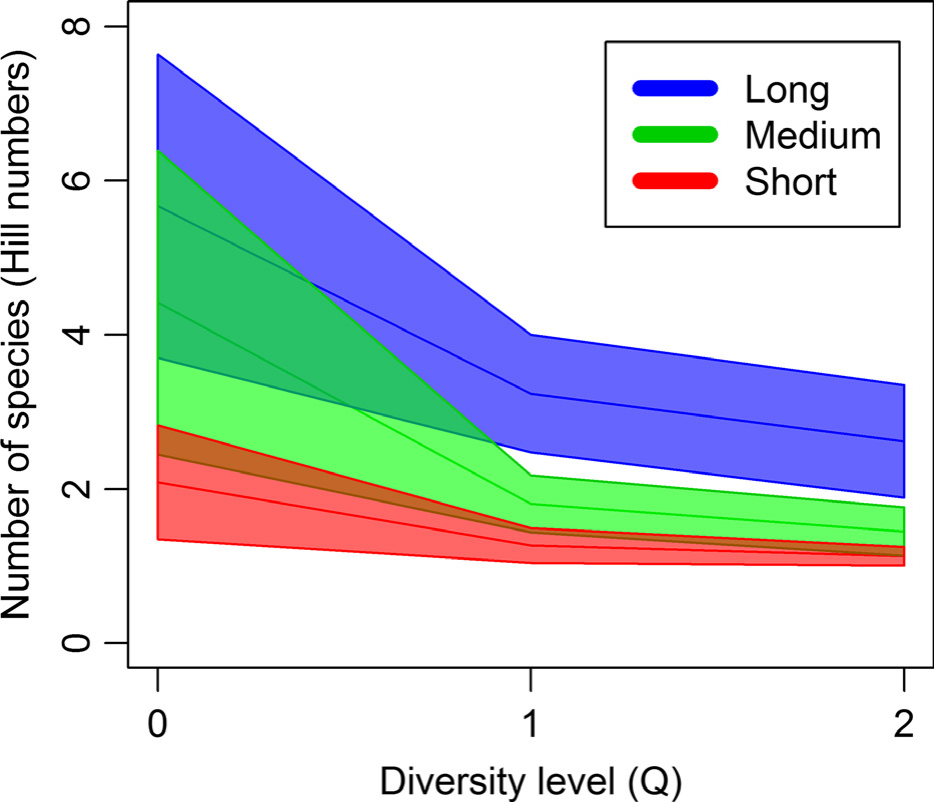
Number of species represented as Hill numbers. Mean values were calculated within each hydroperiod treatment for endpoint communities at three diversity levels, Chao 1 species richness (*q* = 0), estimated exponential Shannon’s index (*q* = 1), and estimated inverse Simpson index (*q* = 2). The colored polygons enclose regions bounded by ±2 SE.

Species richness varied over the duration of the experiment (Fig.5A) and differed significantly between hydroperiod treatments on day 321 (*F* = 9.53, df = 16.92, *P* = 0.002) and day 350, the endpoint community (*F* = 7.12, df = 17.88, *P* = 0.005). On day 321, species richness of the short-hydroperiod treatment was significantly lower than in both the medium (Cohen’s *d* = 1.94, *P* = 0.01)- and long-hydroperiod treatments (Cohen’s *d* = 1.69, *P* = 0.03); on day 350, only the long- and short-hydroperiod treatments differed (Cohen’s *d* = 1.81, *P* = 0.01; Fig.6).

Similar to species richness, Shannon’s index differed between hydroperiod treatments on day 321 (*F* = 8.90, df = 21.34, *P* = 0.002) and day 350 (*F* = 122.58, df = 20.85, *P* < 0.001; Fig.5B). On day 321, the short-hydroperiod treatment was significantly lower than both medium (Cohen’s *d* = 1.62, *P* = 0.005)- and long-hydroperiod treatments (Cohen’s *d* = 1.50, *P* = 0.008), whereas on day 350, the long-hydroperiod treatment had a greater Shannon index than both short (Cohen’s *d* = 3.12, *P* < 0.001)- and medium-hydroperiod treatments (Cohen’s *d* = 1.58, *P* = 0.003; Fig.6).

The Simpson index differed significantly according to hydroperiod treatment on day 321 (*F* = 6.92 df = 21.52, *P* = 0.005) and day 350 (*F* = 29.09, df = 20.90, *P* < 0.001) of the experiment (Fig.5C). The short-hydroperiod treatment differed from both the medium (Cohen’s *d* = 1.38, *P* = 0.02)- and long-hydroperiod (Cohen’s *d* = 1.40, *P* = 0.01) treatments on day 321, whereas the long-hydroperiod treatment differed from both the short (Cohen’s *d* = 3.36, *P* < 0.001)- and medium-hydroperiod (Cohen’s *d* = 1.83, *P* = 0.001) treatments on day 350 (Fig.6). Predation did not have an effect on *α*-diversity on any sampling date.

## Discussion

Prior studies found greater *β*-diversity among permanent ponds than among temporary ponds, implying that *β*-diversity has a positive relationship with hydroperiod (Chase 2003, 2007). However, the present study indicates that this relationship is not simply linear. With respect to species presence–absence, the short-hydroperiod treatment had significantly lower *β*-diversity than the long-hydroperiod treatment, which supports the findings of Chase. However, it was the medium-hydroperiod treatment that had the greatest dissimilarity, indicating that intermediate levels of wetland drying lead to increased *β*-diversity. When dominant species are considered, the pattern followed that implied by Chase (2003, 2007); the long-hydroperiod treatment had the greatest *β*-diversity, followed by the medium, with the short-hydroperiod treatment having the lowest *β*-diversity. This indicates that wetland drying affected rare and dominant species differently. The greater similarity in the two drying treatments suggests that some species respond more favorably to wetland drying and come to dominate the community once wetlands are reflooded.

Hydroperiod also had an important impact on *α*-diversity. Numerous studies in temporary wetland systems have examined the relationship between zooplankton species richness and hydroperiod, with most finding that richness increases with hydroperiod (Serrano and Fahd 2005; Waterkeyn et al. 2008; Boven and Brendonck 2009; Brendonck et al. 2015); however, some studies have found that richness was greatest in wetlands of intermediate hydroperiod (DeBiase and Taylor 2005; Frisch et al. 2006). The present study supports the positive richness-hydroperiod relationship observed in most field studies and also found a positive hydroperiod relationship with both *q*1 and *q*2. The impact of shortened hydroperiod on the higher orders of *α*-diversity is notable, as most studies focused on richness only. Overall, reduced hydroperiods led to communities dominated by just a few abundant and common species, the loss of rarer species, and a relatively low-diversity community.

It should be noted that this experiment did not simply test the effect of hydroperiod length, it also tested drying frequency, and are inseparable in our study design. Hydroperiod length is important because temporary ponds tend to accumulate species as a hydroperiod proceeds, which leads to fewer species present in those that dry earlier (Boven and Brendonck 2009). This accumulation is due to species turnover as niche availability changes. Species can also be excluded from short-hydroperiod ponds if they cannot complete their life cycle during an inundation period (Wellborn et al. 1996). Drying frequency can impact species presence–absence through egg bank depletion; repeated hatchings without egg bank replenishment are known to reduce zooplankton density (Taylor et al. 1990) and could result in species loss or failure to colonize. Both reduced hydroperiod length and increased drying frequency result in lower species richness (Brendonck et al. 2015), and while both processes affected our mesocosms, it is likely that drying frequency in particular was responsible for the reduced diversity observed in the short-hydroperiod treatments.

Predation appeared to have a smaller impact on *β*-diversity than did hydroperiod. While it had no effect on dominant species, it had some effect on the presence of rare species, leading to more similar communities when salamander larvae were present. This result partially supports the prediction that predation should increase community similarity. However, there were no significant differences on *α*-diversity due to predation. This lack of difference suggests that salamander predation, at least at the densities used in this experiment had little effect on diversity, but these effects might have been observed had we used higher predator densities. Studies that have noted effects of salamander larvae predation on zooplankton densities had predator densities that were two to eight times greater than the density we used (Scott 1990; Blaustein et al. 1996). Despite the small effect observed here, mesocosm studies of salamander predation indicate that they can decrease zooplankton density and biomass and increase periphyton, bacteria, and chlorophyll *a* (Scott 1990; Holomuzki et al. 1994; Blaustein et al. 1996). In addition, salamander predation can reduce species richness (Blaustein et al. 1996; Urban 2013), or increase it in situations where two salamander species are present (Urban 2013).

The third prediction that species richness would be greater in intermediate hydroperiod treatments was not supported by the results, although this pattern has been observed in natural wetland systems (DeBiase and Taylor 2005; Frisch et al. 2006). Mean species richness per mesocosm was lower in the medium-hydroperiod treatment than the long-hydroperiod treatment, although this difference was small and not statistically significant. The model that led to this prediction suggested that dissimilarity may be greater in intermediate hydroperiods. Our interpretation was that greater species richness would be the cause. Dissimilarity was indeed greater within the medium-hydroperiod treatment, but species richness was not. Instead, it appears that the greater dissimilarity was the result of differences in species presence/absence between mesocosms.

The predation-permanence gradient model predicts that the processes of predation and demographic constraint due to wetland drying are greatest at opposite ends of the hydrologic gradient (Wellborn et al. 1996). Our data strongly support the latter, but provide only minor support for the former. A reduced set of species was able to persist in the short-hydroperiod treatments, but there were no species unique to the short-hydroperiod mesocosms, whereas a richer assemblage was found in the other two hydroperiod treatments. In contrast, predation had little impact on the experimental communities. However, our experiment was not designed to test increasing intensity of predation as the predation-permanence gradient hypothesizes, but simply whether or not predation could influence diversity in our system. Although our results only found evidence for increased *β*-diversity as measured through species presence–absence, other researchers have found strong impact of salamander predation on species richness (Blaustein et al. 1996; Urban 2013). An extension of the predation-permanence gradient model is that deterministic processes are lessened in intermediate portions of the hydrologic gradient, so that stochastic processes take on greater importance. This was manifested as greater dissimilarity within the medium-hydroperiod treatment instead of differences in species richness as had been anticipated.

A key prediction of the predation-permanence gradient model, the increase in community similarity as hydroperiod is shortened, was supported by this experiment. In addition, shorter hydroperiod communities had lower richness than longer hydroperiod communities. One implication is that a reduction in hydroperiod length among temporary wetlands could lead to a loss of diversity. However, the increased *β*-diversity among intermediate hydroperiod treatments indicates that intermediate levels of dry down may play an important role in maintaining high *γ*-diversity among wetlands.
